# A Novel *De Novo* Assembly Approach for Transcriptome Profiling and Marker Identification in *Grewia Asiatica* (Phalsa), a Medicinal Plant, Across Leaf And Root Tissues

**DOI:** 10.2174/0113892029397989251119050543

**Published:** 2026-04-08

**Authors:** Bharat Taindu Jain, Mukul Jain, Nil Patil, Alpa Yadav, Paramdeep Singh Danda, Meenakshi Sharma, Prashant Kaushik

**Affiliations:** 1 Department of Genetics and Plant Breeding, CCS, HAU, College of Agriculture, Bawal, Haryana 123501, India;; 2 Cell and Developmental Biology Lab, Research & Development Cell, Parul University, Vadodara, Gujarat 391760, India;; 3 Department of Life Sciences, Parul Institute of Applied Sciences, Parul University, Vadodara, Gujarat 391760, India;; 4 Department of Botany, Indira Gandhi University, Rewari 125002, India;; 5 Department of Biochemistry, Punjab Agricultural University, Ludhiana 141027, India;; 6 Department of Chemistry, Ranchi University, Ranchi 834001, India;; 7 Chaudhary Charan Singh Haryana Agricultural University, Hisar 125004, India

**Keywords:** Phalsa, transcriptome, SNPs, SSRs, *de novo* assembly, flavonoid biosynthesis

## Abstract

**Introduction:**

This study aims to conduct a comprehensive transcriptome analysis of *Grewia asiatica* (Phalsa) to identify molecular markers and differential gene expression in leaf and root tissues. *G. asiatica* is recognized for its medicinal properties, attributed to its rich phytochemical profile, including antioxidant, anti-inflammatory, antimicrobial, and hypoglycemic effects. However, genetic insights into the molecular basis of these bioactive properties remain limited.

**Methodology:**

High-throughput Illumina sequencing was performed, generating 32.97 million clean reads from roots and 33.96 million from leaves. A 98.75% complete *de novo* assembly yielded 41,678 unigenes. SNP and SSR markers were identified, and transcription factors across 100 families were characterized. Differential gene expression analysis was conducted, followed by Gene Ontology (GO) and Kyoto Encyclopedia of Genes and Genomes (KEGG) enrichment analyses. Key differentially expressed genes (DEGs) were validated through qRT-PCR.

**Results:**

A total of 316,613 SNPs were identified in roots and 247,967 SNPs in leaves. SSR analysis revealed 21,634 markers, with mononucleotide repeats being predominant. A total of 5,781 transcription factors (TFs) were characterized, including members of the C2H2, MYB-HB-like, and bHLH families. Differential expression analysis identified 4,643 DEGs, with flavonoid biosynthesis genes upregulated in leaves and lignin-associated genes upregulated in roots.

**Discussion:**

GO and KEGG analyses highlighted significant enrichment of metabolic and biosynthetic pathways. The medicinal benefits of *G. asiatica* are linked to its antioxidant properties, which combat oxidative stress; anti-inflammatory effects, which may aid in arthritis relief; antimicrobial activity against pathogens; hypoglycemic effects for blood sugar regulation; and iron content, which is beneficial for anemia treatment.

**Conclusion:**

This study provides valuable SNP and SSR markers and offers insights into the genetic mechanisms underlying the medicinal properties of *G. asiatica*. These findings support future molecular breeding programs and pharmacological research on this underutilized medicinal plant.

## 1 INTRODUCTION

The nutritional and medicinal value of Phalsa (*Grewia asiatica*), a member of the Malvaceae family native to tropical Southeast Asia, is gradually gaining recognition. Traditionally used in Ayurveda, the fruit, leaves, and stems of this plant have been employed to treat a variety of conditions, including inflammation, fever, and digestive disorders [1]. Phalsa is valued not only for its nutritional content but also for its antioxidant properties, whether consumed as whole fruit, juice, or syrup [2]. Nutraceutical research has highlighted numerous pharmacological benefits of Phalsa, including antioxidant activity, antihyperglycemic effects, radioprotective properties, hepatoprotective support, antibacterial, antifungal, analgesic, and antiviral activities. These effects are largely attributed to its rich content of flavonoids, phenolic acids, and anthocyanins, which help prevent chronic diseases by combating oxidative stress [3]. Given the medicinal importance of *G. asiatica*, there is increasing interest in understanding the genetic basis underlying the biosynthesis of its bioactive compounds.

Transcriptome sequencing provides a powerful tool to explore the functional elements of the genome and patterns of gene expression under specific conditions [4]. RNA-seq, in particular, enables the discovery of differentially expressed genes, novel transcripts, and molecular markers. SSRs and SNPs derived from transcriptomes are widely used for genetic mapping, diversity analysis, and molecular breeding in underutilized plants [5-7]. Molecular markers are critical for developing new crop varieties with desirable traits, while analysis of transcription factors (TFs), such as MYB, bHLH, and WRKY, offers insights into the regulation of secondary metabolite biosynthesis [8-10].

In this study, we present a comprehensive transcriptome analysis of leaf and root tissues of *G. asiatica* using a *de novo* assembly approach. Our central hypothesis is that tissue-specific transcriptomic differences underlie the differential biosynthesis of secondary metabolites and can be elucidated through high-throughput sequencing. The primary aim is to construct a reference-quality transcriptome to explore gene expression profiles and identify functional elements involved in secondary metabolism. Specifically, this study seeks to (1) assemble a *de novo* transcriptome of leaf and root tissues, (2) identify and characterize SSR and SNP markers, (3) detect transcription factors involved in biosynthetic regulation, and (4) analyze differentially expressed genes associated with flavonoid and lignin biosynthetic pathways. These findings will provide a valuable genomic resource for functional genomics, marker-assisted breeding, and pharmacological research in this underutilized yet highly valuable medicinal plant.

## 2 MATERIALS & METHODS

### 2.1 Plant Material and RNA Extraction

Healthy root and leaf tissues of *Grewia asiatica* (Phalsa, approximately 10 years old) were collected from Haryana and Fatehabad, India. Immediately after harvesting, the samples were frozen in liquid nitrogen and stored at –80°C until further processing. Total RNA was extracted from each tissue following the manufacturer’s protocol using the Xcelgen Plant RNA Kit (Xcelris Genomics, Bengaluru, India). The quality and quantity of the isolated RNA were assessed using 1% agarose gel electrophoresis, a NanoPhotometer^®^ spectrophotometer (IMPLEN, CA, USA), and the Agilent 2100 Bioanalyzer system with the RNA Nano 6000 Assay Kit (Agilent Technologies, CA, USA). To remove any contaminating genomic DNA prior to library preparation, the RNA samples were treated with RNase-free DNase I (Invitrogen).

### 2.2 RNA Sequencing and Data Processing

After the quality assessment of RNA, 1 µg of RNA per sample was used as the initial material for the RNA sample preparations. The library preparation was performed according to the manufacturer's instructions using the NEBNext^®^ UltraTM RNA Prep Kit for Illumina^®^ (NEB, USA), and index codes were also added to the sequences of the samples under investigation. RNA sequencing libraries with an average length of ~250-300 bp were then sent for sequencing on the Illumina HiSeq™ 2000 platform (Illumina Inc., USA) in the paired-end mode.

### 2.3 *De novo* Transcriptome Assembly

The raw reads were generated using the Illumina HiSeq-2500 paired-end RNA-seq platform, yielding 6.4 Gb for root samples and 6.5 Gb for leaf samples. The raw sequencing data have been submitted to the NCBI-SRA database under BioProject PRJNA540855 (https://www.ncbi.nlm.nih.gov/bioproject/PRJNA540855/). Prior to assembly, raw reads were trimmed to remove adapter sequences, low-quality nucleotides (base quality <20), and poor-quality reads. The quality of the clean reads was assessed by calculating Q20 and Q30 values, GC content, and sequence duplication levels. High-quality reads were then assembled *de novo* using the Trinity RNA-Seq assembler (v. r20140413p1) with a minimum k-mer coverage of 2, while all other parameters were kept at default. The resulting contigs were clustered using Corset v1.05, which groups contigs based on shared reads, separates contigs with distinct expression patterns across samples, and filters out contigs with fewer than 10 mapped reads. The completeness of the *de novo* transcriptome assembly was evaluated using BUSCO v2/v3 through gVolante version 1.2.1 against the plant reference gene set.

### 2.4 SNP, SSR Marker, and Transcription Factor Identification

SNP / Indel mining is one of the most important strategies to understand the variation among plant species. Initially, the raw reads were subjected to Picard tools (v1.41) and Samtools (v0.1.18) to screen, remove duplicated reads, and merge the BAM alignment results of each sample. Then, the Genome Analysis Toolkit (GATK3) software was used to perform SNP mining, and raw VCF files were filtered with the GATK standard filter method, with all other parameters set to default, which is well known for its performance in SNP calling. SNPs and Indel mining were based on the HaplotypeCaller pipeline, which is capable of calling local *de novo* assembly of haplotypes in an active region [12], requiring input in BAM file format for variant calling and generating output as VCF or GVCF files containing raw, unfiltered SNP and Indel information.

Simple Sequence Repeats (SSRs) markers were identified from the Phalsa leaf and root transcriptome using MISA (http://pgrc.ipk-gatersleben.de/misa/misa.html) online web server. The transcripts were checked ten times for mono-repeats, six times for di-repeats, and five times for tri-, tetra-, penta-, and hexa-nucleotide repeats to identify all possible repeats [13].

The *de novo* assembled transcriptome was used to investigate putatively expressed transcription factors using PlantTFcat (http://plantgrn.noble.org/PlantTFcat/). This web-based tool recognizes and categorizes plant transcription factors, proteins/genes, and transcriptional regulators based on relationships between conserved domains and TF genes from 108 published plant TF families. PlantTFcat annotates query sequences with known TF families with high sensitivity and coverage, effectively distinguishing them based on common evolutionary conserved domains [14].

### 2.5 Differential Gene Expression Analysis

After the *de novo* assembly, the transcriptome was filtered using Corset and used as reference sequences for the RSEM software (v1.2.26) [15]. Gene expression levels were estimated by RSEM and measured in Fragments per Kilobase of transcript per Million mapped reads (FPKM). Furthermore, sample correlations, representing the similarity among samples and serving as an important indicator of experimental reliability, were calculated using Pearson’s correlation method.

The read count data obtained from gene expression analysis were normalized using TMM software and subsequently analyzed with DEGSeq (v1.12.0). *P*-values were adjusted using the Benjamini and Hochberg method to control the false discovery rate. Genes with an adjusted *P*-value < 0.05 were considered significantly differentially expressed. Venn diagrams and volcano plots were generated to visualize the overall distribution of differentially expressed genes (DEGs), using thresholds of [log2(Fold Change)] > 1 and q-value < 0.005. Genes exhibiting similar expression patterns were grouped using hierarchical clustering, with different colors representing distinct clusters. In addition to FPKM-based clustering, H-cluster, K-means, and Self-Organizing Map (SOM) methods were applied to cluster the log2-transformed expression ratios.

### 2.6 Functional Annotation and Enrichment Analysis

The obtained unigenes were functionally annotated using the BLAST v2.2.28+ program of the NCBI Nucleotide sequence database (Nt) by the default parameters and filtered with an e-value cutoff of 1e-5. All assembled unigenes were aligned against the non-redundant (Nr) protein database (http://www.ncbi.nlm.nih.gov/), SwissProt (http://www.expasy.ch/sprot/), Kyoto Encyclopedia of Genes and Genomes (KEGG) (http://www.genome.jp/kegg/), and Clusters of Orthologous Groups (COG) (http://www.ncbi.nlm.nih.gov/cog/) databases. Gene ontology (GO) (http://www.geneontology.org/) terms functional annotation was performed based on the best hits from Nr annotation using Blast2go software with an *e-value = 1.0E-6* (http://www.blast2go.de/). After successful GO annotation, the genes were classified into three major gene ontology domains: biological process (BP), cellular component (CC), and molecular function (MF). The DEGs were enriched for GO terms using the R packages GOseq and GOseqR [16]. The KEGG is a priceless database resource for understanding biological systems' functions, molecular information, and applications. We performed statistical enrichment of DEGs in KEGG pathways using the KOBAS program [17].

### 2.7 Quantitative Real-Time PCR (qRT-PCR) Validation

To validate the RNA-Seq data and examine the expression patterns of key transcription factors and genes involved in flavonoid biosynthesis and the phenylpropanoid pathway, quantitative real-time PCR (qRT-PCR) was performed. Total RNA was extracted from leaf and root tissues of *Grewia asiatica* using the Xcelgen Plant RNA Kit (Xcelris Genomics, Bengaluru, India) according to the manufacturer’s instructions. RNA quality and integrity were assessed by 1% agarose gel electrophoresis and quantified using a NanoDrop spectrophotometer (Thermo Fisher Scientific, USA). First-strand cDNA was synthesized from 1 µg of total RNA using the PrimeScript™ RT Reagent Kit with gDNA Eraser (Takara Bio Inc., Japan) following the manufacturer’s protocol. The cDNA was subsequently diluted tenfold with nuclease-free water prior to qRT-PCR reactions.

Gene-specific primers for transcription factors (GaMYB, GabHLH, GaWRKY) and key enzymes in the flavonoid and phenylpropanoid pathways (GaPAL, GaC4H, GaCHS, GaF3H, GaCOMT) were designed based on sequences obtained from the *de novo* assembled transcriptome of *G. asiatica*. Primers were designed using Primer3 software to amplify fragments of 100–200 bp with melting temperatures around 60°C (Table **1**). The GaActin gene was used as the internal control for normalization.

qRT-PCR reactions were performed in a total volume of 20 µL following the manufacturer’s protocol (Tli RNaseH Plus, Takara Bio Inc., Japan). The cycling conditions included an initial denaturation at 95°C for 30 s, followed by 40 cycles of denaturation at 95°C for 5 s and annealing/extension at 60°C for 30 s. A melt curve analysis from 65°C to 95°C was conducted to confirm the specificity of amplification. All reactions were run in triplicate (technical replicates), and three biological replicates were analyzed for each tissue type.

Threshold cycle (Ct) values were obtained using the Bio-Rad CFX Manager software. The relative expression levels of target genes were calculated using the 2−ΔΔCt method [18], with normalization to the internal control gene GaActin. Statistical significance was assessed using a two-tailed Student’s t-test, with differences considered significant at *p* < 0.05.

## 3 RESULTS

### 3.1 Transcriptome Sequencing and Assembly

The HiSeq-2500 Illumina platform generated 43,659,106 and 42,434,450 raw reads for Phalsa leaf and root samples, respectively. After quality filtering, 33,957,116 clean reads (5.1 Gb) for leaf and 32,966,354 clean reads (4.9 Gb) for root were obtained. These reads were assembled *de novo* using Trinity, resulting in 41,678 unigenes. The raw transcriptome reads have been submitted to the NCBI Sequence Read Archive (SRA) under accession numbers SRR10376326 (leaf) and SRR10376325 (root). The average Q30 and GC content were 94.83% and 45.39% for leaf, and 94.64% and 45.59% for root, respectively (Table **2**).

The total assembled length of all transcripts was 43,007,561 bp, while the cumulative length of unigenes was 42,973,118 bp, indicating a high-quality and complete assembly. Trinity further constructed full-length transcripts, yielding 41,893 transcripts and 41,678 unigenes with an average length of 1,027 bp and 1,031 bp, and N50 values of 1,578 bp and 1,580 bp, respectively (Table **3**). The completeness assessment of the transcriptome using BUSCO analysis showed 98.75% of genes were complete. Base composition analysis revealed percentages of A: 28.49%, T: 28.66%, G: 21.33%, and C: 21.53%.

### 3.2 SNP, SSR, and TF Markers Identification

The transcriptome assembly of phalsa revealed a total of 3, 16,613, and 2, 47,967 SNP markers, followed by 7 and 29 insertions, 17 and 15 deletions, 32,968 and 84,314 missing genotypes, and 61751 and 73,031 same as references for root and leaf, respectively (Table **4**).

The Simple Sequence Repeats (SSRs) are highly repetitive nucleotide sequence motifs of lengths ranging between 1–6 base pairs, and they have a wide range of uses, including linkage map development, marker-assisted selection, quantitative trait loci mapping, parentage analysis, genetic diversity studies, gene flow, cultivar fingerprinting, and evolutionary studies [19]. Traditional methods for SSR marker development are costly and labor-intensive, and nowadays transcriptome-wide sequencing has become a viable method for microsatellite mining and for developing SSR-based markers, and has been utilized in many species [20].

Here, we performed phalsa leaf and root transcriptome assembly and identified SSRs using the MISA web tool. Among all the identified 21,634 SSR microsatellites, the mononucleotide type was found to be the highest, with 11,908 recorded, followed by 4,744 (Dinucleotide), 4,383 (Trinucleotide), 1,508 (Tetranucleotide), 725 (Pentanucleotide), and 462 (Hexanucleotide). The total length of SSRs was 37,240 bp, with relative abundance and density of 260.85 SSR/Mb and 449.02 bp/Mb, respectively. The graphical presentation of all detected SSRs and SSR types is shown in Fig. **1**.

Transcription Factors regulate gene expression, and they possess DNA-binding domains (DBDs) that can recognize specific DNA sequences to mediate the TF–DNA interaction and can activate or suppress the transcription of their target genes to precisely control cell phenotypes and functions [21]. We used the PlantTFcat tool to identify expressed transcription factors based on the signature domain of TF families.

In this study, a total of 5,781 transcription factor genes were identified, belonging to 100 known TF families in the *G. asiatica* leaf and root transcriptome assembly. Among the predicted TFs, the major family was C2H2 (918), followed by WD40-like (645), MYB-HB-like (297), PHD (269), CCHC (Zn) (244), bHLH (203), AP2-EREBP (166), bZIP (187), C3H (173), GRAS (109), Homobox-Wox (116), NAM (136), and WRKY (144) (Fig. **2**).

### 3.3 Functional Annotation and Classification of Unigenes

Forty-one thousand six hundred seventy-eight unigenes were obtained with annotated information, of which 31,534 (75.66%), 27,836 (66.78%), 12,221 (29.32%), 24,273 (58.23%), 22,000 (52.78%), 22,092 (53%), 10,856 (26.04%), and 6,127 unigenes (14.07%) were annotated in all seven databases (Table **3**). The high percentage of *G. asiatica* sequences was closely matched with Theobroma cacao (69.1%), followed by Gossypium raimondii (13.2%), Gossypium arboreum (4.0%), and others (12.1%). The highest unigenes were assigned to the cellular process, followed by the metabolic process in Biological Process, and in the Cellular Component group, a large number of unigenes were assigned to the cell, followed by the cell part, and the highest unigenes were involved in the binding category, followed by catalytic activity in molecular functions (Fig. **3**).

The KOG classification further divided these unigenes into 26 groups based on their involvement and reported high contribution in General function followed by Post-translational modification and Translation, ribosomal structure and biogenesis (Fig. **4**). Similarly, all annotated unigenes were assigned to their associated KEGG pathways in five major phenomena, including Cellular Processes (540), Environmental Information Processing (409), Genetic Information Processing (2748), Metabolism (5590), and Organismal Systems (512) (Fig. **5**).

### 3.4 Differential Gene Expression Analysis

The differential gene expression was measured by estimating individual transcript abundance by mapping the high-quality cleaned reads back to the *de novo* assembled transcripts using RSEM, and their expression levels were recorded as FPKM values (Fig. **6A** & **B**). To determine the relationship between samples, the Pearson correlation was obtained in a range of 0.12 (acceptable range is -1.0 to 1.0) for each sample, which indicates a positive relationship among the analysed samples, whereas gene expression level was compared through the FPKM expression density scatter graph in leaf and roots (Fig. **6C**). 89.02% (30,229,456) reads were aligned from leaf, and 85.06% (28,040,368) reads from roots in comparison to 33,957,116 (leaf) and 32,966,354 (root) original clean reads, respectively (Table **5**).

We found that 23,879 unigenes were commonly expressed in the Leaf and root samples of *G. asiatica*, whereas 11,387 unigenes were specifically expressed in the root, and 3,892 unigenes were specific to the leaf. According to the relative expression levels between leaf and root, the DEGs were analyzed into up-regulated and down-regulated genes. A total of 4,643 DEGs were identified between leaf and root, of which 2,541 and 2,102 were up-regulated and down-regulated, respectively, while a Volcano plot was generated using log2 fold-change against log10 (*p*-value), exhibiting the statistically significant differences used to deduce the overall distribution of differentially expressed genes (Fig. **7A** & **B**). Differentially Expressed Genes were clustered using their log10 (FPKM+1) values, with highly expressed genes denoted with a red pattern, and the blue pattern denoting the lower expression of the genes. The red and blue colour range represents the log10 (FPKM+1) value from large to small (Fig. **7C**). Each grey line in a subline chart represents the relative expression value of a gene cluster under different experimental conditions, and the blue line represents the mean value. Additionally, the Log2 (ratios) line chart for clustered genes was also illustrated for all 4,643 clustered DEGs and divided into 8 sub-clusters, distributing 970, 1,070, 764, 653, 520, 390, 165, and 111 genes to each sub-cluster, respectively, as per their relative expression (Fig. **7D**).

### 3.5 GO Enrichment and KEGG Enrichment Analysis of DEGs

In particular, upregulated and downregulated DEGs were significantly enriched in the BP domain, with the highest enrichment for metabolic process, followed by biosynthetic process and oxidation-reduction process (Fig. **8A** & **B**). The KEGG enrichment of the DEGs generated a scattered plot for the number of genes enriched in the associated pathway. The top 20 significantly DEGs enriched pathways are displayed in the report (Fig. **8C**).

### 3.6 qRT-PCR Analysis

The qRT-PCR analysis revealed significant differences in the expression of transcription factors and key enzymes involved in flavonoid biosynthesis between leaf and root tissues (Supplementary Datasheet **S1**) (Fig. **9**). Notably, the transcription factors *GaMYB* and *GabHLH* were found to be significantly upregulated in leaf tissues compared to roots. Specifically, *GaMYB* expression in leaves was approximately 9.5-fold higher than in roots (*p* < 0.01), while *GaHLH* expression was about 8.1-fold higher in leaves (*p* < 0.01). Additionally, *GaWRKY* exhibited a moderate increase in leaves, with a 3.2-fold higher expression compared to roots (*p* < 0.05).

In terms of flavonoid biosynthesis, key enzymes displayed markedly higher expression levels in leaves. *GaPAL* (Phenylalanine ammonia-lyase) was upregulated by 12.4-fold in leaves (*p* < 0.01), and *GaC4H* (Cinnamate 4-hydroxylase) showed a 10.2-fold increase (*p* < 0.01). Furthermore, *GaCHS* (Chalcone synthase) expression was 7.8-fold higher in leaves (*p* < 0.01), and *GaF3H* (Flavanone 3-hydroxylase) had a significant upregulation of 6.5-fold in leaves (*p* < 0.01). Conversely, the phenylpropanoid pathway gene *GaCOMT* (Caffeic acid O-methyltransferase) exhibited significantly higher expression in roots, with a 5.7-fold increase compared to leaves (*p* < 0.01). These findings underscore the distinct regulatory mechanisms governing gene expression in different plant tissues, particularly concerning flavonoid biosynthesis and related pathways.

## 4 DISCUSSION

Grewia asiatica (Phalsa) is well-known for its nutritious fruits and medicinal properties. The fruit and leaves have been found to possess antioxidant, anticancer, radioprotective, and anti-hyperglycemic effects, while the stem bark demonstrates analgesic and anti-inflammatory characteristics [22]. Due to its phytochemical richness, G. asiatica has been extensively utilized in traditional medicine across Asia, particularly in the prevention and treatment of diseases [23]. The leaves and stems are even used to treat malaria and rheumatism [24]. Despite the growing interest in G. asiatica, its genome remains unavailable in public databases like NCBI, emphasizing the importance of transcriptome data to advance molecular research in this species.

In this study, we conducted the first transcriptome-wide research on G. asiatica using RNA sequencing technologies, resulting in high-quality transcriptomic data. After filtering poor-quality reads, we obtained 33,957,116 clean reads from leaves and 32,966,354 from roots. The de novo assembly of these reads yielded 41,893 transcripts and 41,678 unigenes, with BUSCO analysis indicating a 98.75% completeness, confirming the high-quality nature of the assembly [25]. This transcriptome data provided valuable resources for functional annotation, revealing 41,893 assembled transcripts categorized into molecular function, biological processes, and cellular components using Gene Ontology (GO) analysis. Similar annotations have been performed in other medicinal plants, such as Zanthoxylum armatum, confirming the consistency and reliability of the approach [26]. Furthermore, KEGG pathway analysis linked identified unigenes to critical metabolic and biosynthetic pathways, as well as processes like hormone signal transduction and plant-pathogen interactions, as previously seen in Hedera helix [27].

A major focus of this study was to identify Single Nucleotide Polymorphisms (SNPs) and Simple Sequence Repeats (SSRs), which serve as important molecular markers. Our analysis identified 316,613 SNPs and 247,967 SSRs using the GATK3 tool. Among the identified SSRs, mononucleotide repeats were the most abundant (55%), followed by di- and tri-nucleotide repeats, contributing to an SSR density of 449.02 bp/Mb [28]. These markers offer valuable resources for future genetic diversity and population structure studies in *G. asiatica* and related species. SSR markers are particularly useful in phylogeographic research, forensic science, population genetics, and molecular breeding [29], and their identification here marks a significant contribution to the study of *G. asiatica*. Moreover, this study identified 5,781 transcription factors (TFs) from the transcriptome data, classified into 100 different families, including C2H2, WD40, MYB-HB-like, and WRKY. These transcription factors play crucial roles in regulating various metabolic and developmental processes, including secondary metabolite production. TFs such as MYB and bHLH are known to regulate flavonoid biosynthesis, a key pathway in many medicinal plants [30]. The WRKY TFs, among others, are involved in stress responses and secondary metabolite biosynthesis, further highlighting their importance in medicinally significant pathways [31, 32]. Our findings align with previous studies in other plants, such as Hedera helix and Arundo donax, confirming that transcription factors are essential for controlling secondary metabolism at both transcriptional and translational levels [33].

The qRT-PCR results corroborate the RNA-Seq data, confirming the differential expression of key transcription factors and biosynthetic genes between leaf and root tissues in *Grewia asiatica*. The significant upregulation of GaMYB and GabHLH transcription factors in leaves aligns with their established roles in activating genes involved in flavonoid biosynthesis [32]. Flavonoids play crucial roles in plant defense, UV protection, and contribute to the antioxidant properties of medicinal plants [34]. The elevated expression of flavonoid biosynthetic genes (GaPAL, GaC4H, GaCHS, and GaF3H) in leaves suggests that this tissue is a primary site for flavonoid accumulation in *G. asiatica*. These findings support the traditional use of phalsa leaves in herbal remedies for their health-promoting properties. The higher expression of GaCOMT in roots indicates a specialized function in root metabolism, potentially related to lignin biosynthesis and structural integrity [35]. Lignin is a key component of the plant cell wall and is important for water transport and defense against pathogens [36]. The coordinated expression patterns observed highlight the complex regulatory networks governing secondary metabolite production in *G. asiatica*. Understanding these pathways provides valuable insights into the molecular basis of the plant's medicinal properties and offers potential targets for metabolic engineering to enhance the production of beneficial compounds. This research marks a significant advancement in the molecular study of *G. asiatica* and opens avenues for further investigations into its genetic and pharmacological potential.

## 5 FUTURE PERSPECTIVE

This study establishes a basic transcriptomic structure for *Grewia asiatica*, a plant with important medicinal uses and limited genomic research. The presence of 41,000 unigenes, 565,000 SNPs in tissues, and 21,000 SSR markers in the dataset provides a solid set of tools for research. Genetic diversity can be studied in detail, new traits may be linked to markers, and association studies involving the entire genome may be performed with these resources in *G. asiatica* and close relatives. Efforts in the next steps should consider the gene functions of GaMYB, GabHLH, and GaCOMT, which are closely linked to the biosynthesis of flavonoids and the formation of lignin. Combining transcriptomic data with metabolomics and epigenomics results will clarify how secondary metabolites are produced, which will help in targeting metabolic engineering. Since flavonoids and phenolics are important in pharmacology, improving metabolic pathways with CRISPR/Cas9 or RNAi technology could result in new treatments from plants. Moreover, having a reference genome for *G. asiatica* would improve transcriptome annotation and allow for more comparative genomics within the Malvaceae family. Carrying out multi-genotype and multi-stress transcriptomic studies can help find changes in genes that allow organisms to handle stress. Developing hairy root or callus cultures could be useful for improving the production of desired metabolites. By using both systems and synthetic biology, this can speed up moving genomic findings into applications, creating common herbal products as well as improved crop cultivars. Overall, this research sets up *G. asiatica* to serve as a genomic model for medicinal plants, helping with developments in molecular breeding, phytomedicine, and ecologically friendly agro-biotechnology.

## CONCLUSION

In this study, we successfully conducted the first comprehensive transcriptome analysis of *Grewia asiatica* (Phalsa), a nutritionally and medicinally important plant. By utilizing next-generation sequencing (NGS) and *de novo* transcriptome assembly, we generated high-quality transcriptomic data from both leaf and root tissues. The resulting assembly revealed 41,678 unigenes with a completeness score of 98.75%, as assessed by BUSCO, affirming the reliability of the data. Our analysis identified a total of 316,613 SNPs and 247,967 SSRs, which represent valuable molecular markers for future studies on genetic diversity, population structure, and marker-assisted breeding in *G. asiatica* and related species. The identified SNPs and SSRs also hold potential for linking specific genetic variations to the plant’s bioactive compounds, furthering its use in pharmacological applications. Additionally, we identified 5,781 transcription factors (TFs) belonging to 100 different families, including key regulatory families such as C2H2, WD40, MYB-HB-like, and WRKY. These TFs play critical roles in controlling gene expression, metabolic pathways, and the biosynthesis of secondary metabolites, highlighting their significance in the medicinal properties of *G. asiatica*. Overall, this study provides a strong transcriptome resource for *G. asiatica*, facilitating gene discovery, functional analyses, and molecular breeding efforts. The dataset generated here lays the groundwork for deeper explorations into the molecular basis of its therapeutic potential and its adaptation to environmental stresses. Future research can build upon these findings to explore the genetic regulation of its bioactive compounds and further unlock the genetic and pharmacological potential of *G. asiatica*.

## Figures and Tables

**Fig. (1):**
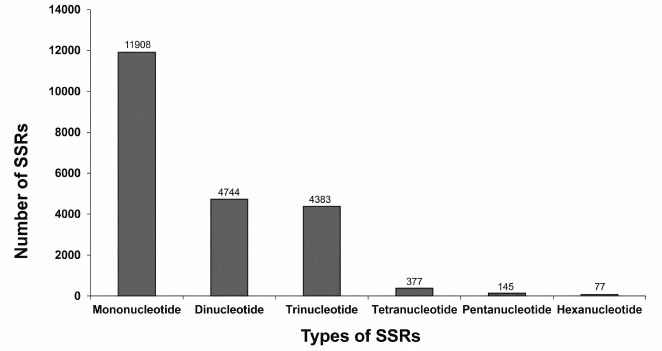
SSR molecular marker distribution in phalsa root and leaf transcriptome assembly.

**Fig. (2):**
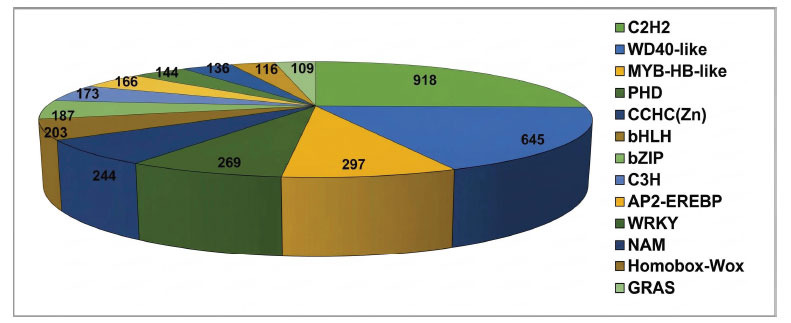
Graphical presentation of major transcription factor families in phalsa leaves and root assembly.

**Fig. (3):**
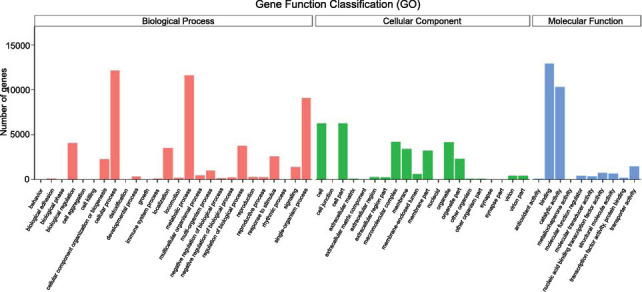
Functional classification of unigenes, Gene Ontology categorization under three main GO domains (BP, CC, and MF).

**Fig. (4):**
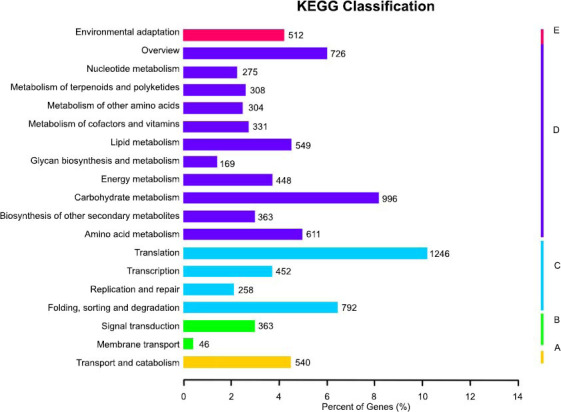
Functional classifications of unigenes, clusters of orthologous group (COG) classification of unigenes, and grouped into 26 COG classes.

**Fig. (5):**
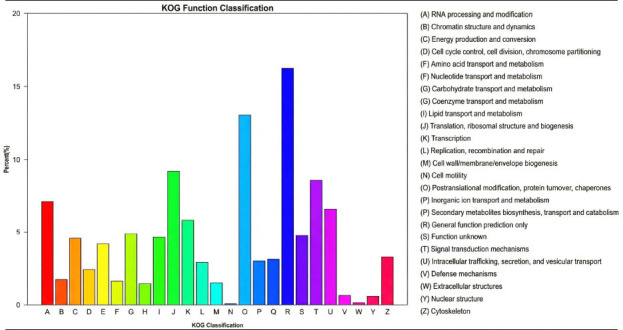
The KEGG metabolic pathways showing five branches for involved unigenes: **A**: Cellular Processes, **B**: Environmental Information Processing, **C**: Genetic Information Processing, **D**: Metabolism, **E**: Organismal Systems.

**Fig. (6):**
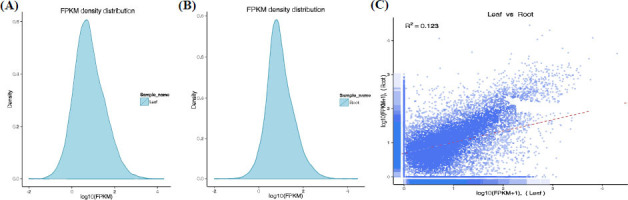
Gene Expression analysis, (**A**) FPKM density distribution in leaf, (**B**) FPKM density distribution in root, and (**C**) FPKM values comparison between leaf and root samples.

**Fig. (7):**
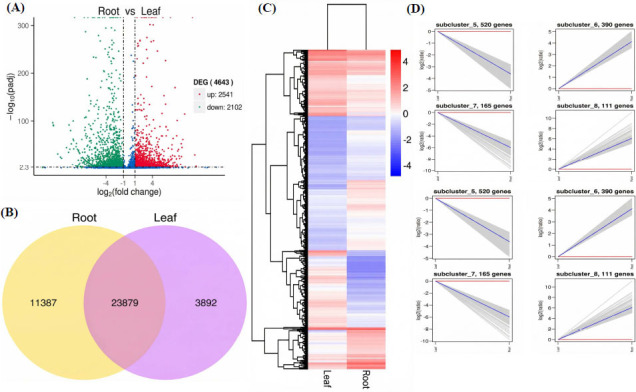
Analysis of DEGs between leaf and root (**A**) Volcano plat for the difference the gene expression levels was plotted for the leaf library *vs*. the root library, (**B**) Venn diagram demonstrating the number of unigenes expressed in Phalsa between root and leaf, (**C**) Results of FPKM cluster analysis for leaf and root of Phalsa, (**D**) Log2 (ratios) line chart for clustered DEGs.

**Fig. (8):**
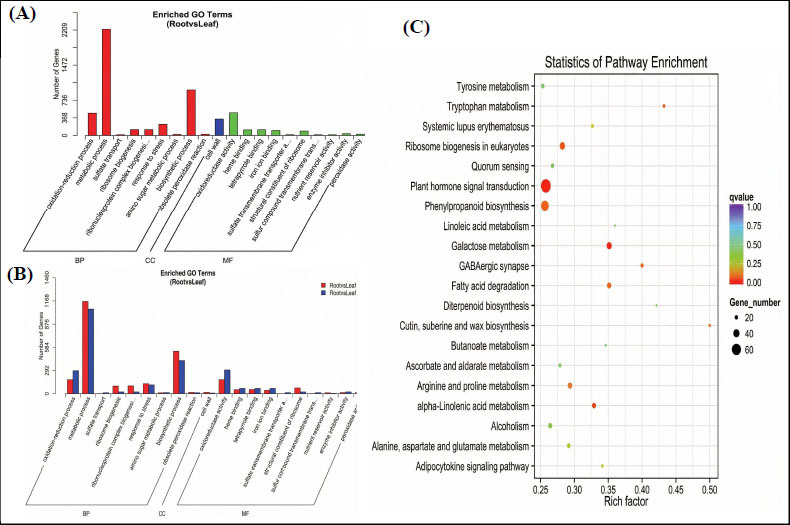
(**A**) Gene Ontology analysis for all DEGs, assigned three main domains, (**B**) GO enrichment-based statistical histogram of up-regulated and down-regulated genes, (**C**) KEGG enrichment of DEGs in the top 20 pathways.

**Fig. (9):**
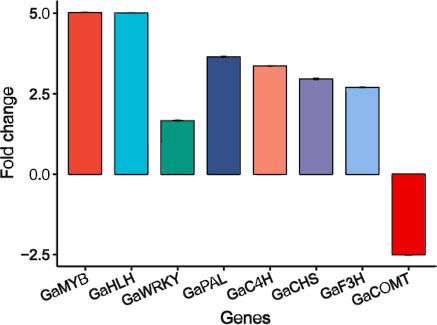
Relative expression levels of selected genes in leaf and root tissues of *Grewia asiatica*. Expression levels were normalized to *GaActin* and calculated using the *2^−ΔΔCt^* method (plotted with SRPlot).

**Table 1:** Primers used for qRT-PCR analysis.

**Gene**	**Primer Name**	**Primer Sequence (5'→3')**	**Product Size (bp)**
GaMYB	GaMYB-F	ATGGTGCTGCTGATGCTGATG	158
	GaMYB-R	CAGGATGAGGGTGAGGAGGAG	
GabHLH	GabHLH-F	GATGCTGCTGGTGGTGATGGT	142
	GabHLH-R	CGATGAGGAGGAGGAGGAAGA	
GaWRKY	GaWRKY-F	AGGAGGAGGAGGAGGAGGAG	130
	GaWRKY-R	TCTGGTGGTGGTGGTGGTGTA	
GaPAL	GaPAL-F	ATGGAGGAGGAGGAGGAGGAG	155
	GaPAL-R	TCCTCCAGGAGGAGGAGGAGT	
GaC4H	GaC4H-F	GCTGCTGCTGCTGCTGCTGCT	148
	GaC4H-R	GAGGAGGAGGAGGAGGAGGA	
GaCHS	GaCHS-F	ATGGCTGCTGCTGCTGCTGCT	162
	GaCHS-R	GAGGAGGAGGAGGAGGAGGA	
GaF3H	GaF3H-F	CTGCTGCTGCTGCTGCTGCT	138
	GaF3H-R	GAGGAGGAGGAGGAGGAGGA	
GaCOMT	GaCOMT-F	ATGGCTGCTGCTGCTGCTGCT	150
	GaCOMT-R	CAGGAGGAGGAGGAGGAGGAG	
GaActin	GaActin-F	ATGGCTGCTGCTGCTGCTGCT	145
	GaActin-R	TCGATGAGGAGGAGGAGGAGG	

**Table 2:** Statistical summary of the *de novo* assembly for *G. asiatica*.

**Sample**	**Raw Reads**	**Clean Reads**	**Clean Bases**	**Error (%)**	**Q20 (%)**	**Q30 (%)**	**GC (%)**
Leaf	43,659,106	33,957,116	5.1G	0.01	97.95	94.83	45.39
Root	42,434,450	32,966,354	4.9G	0.01	97.84	94.64	45.59

**Table 3:** Summary of the number of transcripts and unigenes in different length intervals and length distribution.

**Categories**	**200-500bp**	**500-1kbp**	**1k-2kbp**	**>2kbp**	**Min Length**	**Mean Length**	**Max Length**	**N50**	**N90**	**Total**
Transcripts	15,713	10,288	10,432	5,460	201	1,027	10,772	1,578	444	41,893
Unigenes	15,499	10,287	10,432	5,460	201	1,031	10,772	1,580	447	41,678

**Table 4:** Summary of SNP mining from phalsa root and leaf using GATK3.

**Factors**	**Root**	**Leaf**
SNPs	316613	247967
MNPs	0	0
Insertions	7	29
Deletions	17	15
Indels	0	0
Same as reference	61751	79031
Missing Genotype	32968	84314
SNP Transitions / Transversions	1.64 (226743/138266)	1.64 (178242/108893)
Total Het / Hom ratio	5.54 (268241/48396)	5.33 (208838/39173)
SNP Het / Hom ratio	5.54 (268217/48396)	5.33 (208799/39168)
MNP Het / Hom ratio	(0/0)	(0/0)
Insertion Het / Hom ratio	(7/0)	6.25 (25/4)
Deletion Het / Hom ratio	(17/0)	14.00 (14/1)
Indel Het/Hom ratio	(0/0)	(0/0)
Insertion/Deletion ratio	0.41 (7/17)	1.93 (29/15)
Indel/SNP+MNP ratio	0.00 (24/316613)	0.00 (44/247967)

**Table 5:** Alignment overview of the reads for gene expression analysis.

**Sample Name**	**Total Reads**	**Total Mapped**	
Leaf	33,957,116		30,229,456 (89.02%)
Root	32,966,354	28,040,368 (85.06%)	-

## Data Availability

The authors confirm that the data supporting the findings of this research are available within the article.

## LIST OF ABBREVIATIONS

BP: Biological Process
CC: Cellular Component
COG: Clusters of Orthologous Groups
FPKM: Fragments Per Kilo Base of Transcript Per Million Mapped Reads
GATK3: Genome Analysis Tool Kit
GO: Gene Ontology
KEGG: Kyoto Encyclopedia of Genes and Genomes
MF: Molecular Function
SSRs: The Simple Sequence Repeats
Tfs: Transcription Factors
