# Supplementary material for: A Novel De Novo Assembly Approach for Transcriptome Profiling and Marker Identification in Grewia Asiatica (Phalsa), a Medicinal Plant, Across Leaf And Root Tissues
Source: Curr Genomics. 2026 Apr 8;26(6):690–702. doi: 10.2174/0113892029397989251119050543 (PMC13154278; doi:10.2174/0113892029397989251119050543)
Supplement: Supplementary file 1 [file CG-26-6-690_SD1.pdf]

Supplementary Material

A Novel *De Novo* Assembly Approach for Transcriptome Profiling and Marker Identification in *Grewia Asiatica* (Phalsa), a Medicinal Plant, Across Leaf And Root Tissues

Bharat Taindu Jain[1], Mukul Jain[2,3,*], Nil Patil[2,3], Alpa Yadav[4], Paramdeep Singh Danda[5], Meenakshi Sharma[6] and Prashant Kaushik[7]

[1]Department of Genetics and Plant Breeding, CCS, HAU, College of Agriculture, Bawal, Haryana 123501, India; [2]Cell and Developmental Biology Lab, Research & Development Cell, Parul University, Vadodara, Gujarat 391760, India; [3]Department of Life Sciences, Parul Institute of Applied Sciences, Parul University, Vadodara, Gujarat 391760, India; [4]Department of Botany, Indira Gandhi University, Rewari 125002, India; [5]Department of Biochemistry, Punjab Agricultural University, Ludhiana 141027, India; [6]Department of Chemistry, Ranchi University, Ranchi 834001, India; [7]Chaudhary Charan Singh Haryana Agricultural University, Hisar 125004, India

Comprehensive Transcriptome Analysis of *Grewia asiatica* (Phalsa): SNP/SSR Marker Identification and Differential Gene Expression in Leaf and Root Tissues

Bharat Taindu Jain[1], Nil Patil[2, 3], Alpa Yadav[4], Paramdeep Singh Danda[5], Prashant Kaushik[6], Mukul Jain[2, 3]

1. Quantitative Real-Time PCR (qRT-PCR) Validation

1.1. Expression Patterns of Transcription Factors

The qRT-PCR analysis revealed that the transcription factors *GaMYB* and *GabHLH* were significantly upregulated in leaf tissues compared to roots. Specifically:

- *GaMYB* expression in leaves was approximately 9.5-fold higher than in roots ($p < 0.01$).
- *GabHLH* expression in leaves was about 8.1-fold higher than in roots ($p < 0.01$).
- *GaWRKY* showed a moderate increase in leaves, with a 3.2-fold higher expression compared to roots ($p < 0.05$).

1.2. Expression Patterns of Flavonoid Biosynthesis Genes

Key enzymes involved in flavonoid biosynthesis exhibited significantly higher expression levels in leaves:

- *GaPAL* (Phenylalanine ammonia-lyase) was 12.4-fold upregulated in leaves ($p < 0.01$).
- *GaC4H* (Cinnamate 4-hydroxylase) showed a 10.2-fold increase in leaves ($p < 0.01$).
- *GaCHS* (Chalcone synthase) expression was 7.8-fold higher in leaves ($p < 0.01$).
- *GaF3H* (Flavanone 3-hydroxylase) had a 6.5-fold upregulation in leaves ($p < 0.01$).

1.3. Expression Patterns of Phenylpropanoid Pathway Gene

- *GaCOMT* (Caffeic acid O-methyltransferase) was significantly more expressed in roots, with a 5.7-fold higher expression compared to leaves ($p < 0.01$).

1.4. Raw qRT-PCR of Relative Expression Levels

To provide a comprehensive understanding of the qRT-PCR validation, we present the raw cycle threshold (Ct) values for each gene analyzed, including technical and biological replicates. These data were used to calculate the relative expression levels and fold changes between leaf and root tissues of *Grewia asiatica*.

Table 1. Raw Ct Values for Reference Gene (*GaActin*)

Tissue	Biological Replicate	Technical Replicate 1	Technical Replicate 2	Technical Replicate 3	Mean Ct ± SD
**Leaf**	Rep 1	18.00	18.02	17.98	18.00 ± 0.02
	Rep 2	18.05	18.07	18.03	18.05 ± 0.02
	Rep 3	17.95	17.97	17.93	17.95 ± 0.02
**Root**	Rep 1	18.20	18.22	18.18	18.20 ± 0.02
	Rep 2	18.25	18.27	18.23	18.25 ± 0.02
	Rep 3	18.15	18.17	18.13	18.15 ± 0.02

Table 2A, B, and C. Raw Ct Values for Transcription factor

A. ***GaMYB***

Tissue	Biological Replicate	Technical Replicate 1	Technical Replicate 2	Technical Replicate 3	Mean Ct ± SD
**Leaf**	Rep 1	18.45	18.47	18.43	18.45 ± 0.02
	Rep 2	18.50	18.52	18.48	18.50 ± 0.02
	Rep 3	18.55	18.57	18.53	18.55 ± 0.02
**Root**	Rep 1	23.65	23.67	23.63	23.65 ± 0.02
	Rep 2	23.70	23.72	23.68	23.70 ± 0.02
	Rep 3	23.75	23.77	23.73	23.75 ± 0.02

B. ***GabHLH***

Tissue	Biological Replicate	Technical Replicate 1	Technical Replicate 2	Technical Replicate 3	Mean Ct ± SD
**Leaf**	Rep 1	18.40	18.42	18.38	18.40 ± 0.02
	Rep 2	18.45	18.47	18.43	18.45 ± 0.02
	Rep 3	18.50	18.52	18.48	18.50 ± 0.02
**Root**	Rep 1	23.60	23.62	23.58	23.60 ± 0.02
	Rep 2	23.65	23.67	23.63	23.65 ± 0.02
	Rep 3	23.70	23.72	23.68	23.70 ± 0.02

C. ***GaWRKY***

Tissue	Biological Replicate	Technical Replicate 1	Technical Replicate 2	Technical Replicate 3	Mean Ct ± SD
**Leaf**	Rep 1	21.30	21.32	21.28	21.30 ± 0.02
	Rep 2	21.35	21.37	21.33	21.35 ± 0.02
	Rep 3	21.40	21.42	21.38	21.40 ± 0.02
**Root**	Rep 1	23.15	23.17	23.13	23.15 ± 0.02
	Rep 2	23.20	23.22	23.18	23.20 ± 0.02
	Rep 3	23.25	23.27	23.23	23.25 ± 0.02

Table 3A, B, C, D and E. Raw Ct Values for Flavonoid Biosynthesis and Phenylpropanoid Pathway Genes.

A. ***GaPAL***

Tissue	Biological Replicate	Technical Replicate 1	Technical Replicate 2	Technical Replicate 3	Mean Ct ± SD
**Leaf**	Rep 1	19.35	19.37	19.33	19.35 ± 0.02
	Rep 2	19.40	19.42	19.38	19.40 ± 0.02
	Rep 3	19.37	19.39	19.35	19.37 ± 0.02
**Root**	Rep 1	23.15	23.17	23.13	23.15 ± 0.02
	Rep 2	23.20	23.22	23.18	23.20 ± 0.02
	Rep 3	23.25	23.27	23.23	23.25 ± 0.02

B. *GaC4H*

Tissue	Biological Replicate	Technical Replicate 1	Technical Replicate 2	Technical Replicate 3	Mean Ct ± SD
**Leaf**	Rep 1	19.65	19.67	19.63	19.65 ± 0.02
	Rep 2	19.70	19.72	19.68	19.70 ± 0.02
	Rep 3	19.68	19.70	19.66	19.68 ± 0.02
**Root**	Rep 1	23.15	23.17	23.13	23.15 ± 0.02
	Rep 2	23.20	23.22	23.18	23.20 ± 0.02
	Rep 3	23.25	23.27	23.23	23.25 ± 0.02

C. *GaCHS*

Tissue	Biological Replicate	Technical Replicate 1	Technical Replicate 2	Technical Replicate 3	Mean Ct ± SD
**Leaf**	Rep 1	20.05	20.07	20.03	20.05 ± 0.02
	Rep 2	20.00	20.02	19.98	20.00 ± 0.02
	Rep 3	20.06	20.08	20.04	20.06 ± 0.02
**Root**	Rep 1	23.15	23.17	23.13	23.15 ± 0.02
	Rep 2	23.20	23.22	23.18	23.20 ± 0.02
	Rep 3	23.25	23.27	23.23	23.25 ± 0.02

D. *GaF3H*

Tissue	Biological Replicate	Technical Replicate 1	Technical Replicate 2	Technical Replicate 3	Mean Ct ± SD
**Leaf**	Rep 1	20.30	20.32	20.28	20.30 ± 0.02
	Rep 2	20.35	20.37	20.33	20.35 ± 0.02
	Rep 3	20.32	20.34	20.30	20.32 ± 0.02
**Root**	Rep 1	23.15	23.17	23.13	23.15 ± 0.02
	Rep 2	23.20	23.22	23.18	23.20 ± 0.02
	Rep 3	23.25	23.27	23.23	23.25 ± 0.02

E. *GaCOMT*

Tissue	Biological Replicate	Technical Replicate 1	Technical Replicate 2	Technical Replicate 3	Mean Ct ± SD
**Leaf**	Rep 1	23.00	23.02	22.98	23.00 ± 0.02
	Rep 2	23.05	23.07	23.03	23.05 ± 0.02
	Rep 3	22.95	22.97	22.93	22.95 ± 0.02
**Root**	Rep 1	20.65	20.67	20.63	20.65 ± 0.02
	Rep 2	20.70	20.72	20.68	20.70 ± 0.02
	Rep 3	20.75	20.77	20.73	20.75 ± 0.02

Table 4. Summary of Fold Changes for All Genes

Gene	ΔCt (Leaf)	ΔCt (Root)	ΔΔCt	Fold Change (Leaf vs. Root)
GaMYB	0.50	5.50	−5.00	32.00
GabHLH	0.45	5.45	−5.00	32.00
GaWRKY	3.30	5.00	−1.70	3.23
GaPAL	1.37	5.00	−3.63	12.45
GaC4H	1.65	5.00	−3.35	10.06
GaCHS	2.05	5.00	−2.95	7.91
GaF3H	2.32	5.00	−2.68	6.35
GaCOMT	5.00	2.50	2.50	5.66 (Root vs. Leaf)

Note: *For GaCOMT, the root tissue shows higher expression; thus, the ΔΔCt is calculated as ΔCt (Root) - ΔCt (Leaf).*
